# Medical professionalism in Lebanon: between doctors' perception and patients' satisfaction

**DOI:** 10.5116/ijme.5f28.77a3

**Published:** 2020-08-21

**Authors:** Carole Saade Riachy, Elie Nemr

**Affiliations:** 1 Department of Pedagogy, Faculty of Medicine, Saint Joseph University, Beirut, Lebanon

**Keywords:** Medical professionalism, Lebanon, doctors' perception, patients' satisfaction

## To the Editor

Medical professionalism is the foundation of the social contract between the medical profession and society, and observable conduct that reflects the values and norms of the medical profession.^1^

The doctor's role keeps evolving as society changes, and has shifted to more collaboration with patients; it is currently the relationship hallmark.^2^ The doctor-patient relation is described as "a consensual relationship where the patient knowingly requires the assistance of the doctor and where the doctor knowingly recognizes the individual as a patient".^3^ This relationship is a sign of integrity and the cornerstone of quality health care.^4^ "Patient satisfaction" is a legitimate outcome and an essential condition for the continuation of the therapy.^5^

In Canada, the Quebec Medical Association (QMA) has launched the first milestone that includes a reflection and an action plan under the theme "The Medical Profession: Towards a New Social Contract".^6^ This idea led to a reflection on the future of the medical profession in Lebanon and its relationship with the society. The aim was to understand the perception of the doctor's status in the health system and society.

To the best of our knowledge, this study is the first in Lebanon to examine the general public's perception of doctors from a societal perspective and the doctor' opinions towards their patients. The sample included 50 Lebanese doctors and 171 Lebanese adults of the general population who visited at least one doctor in their lifetime. The questionnaire was based on  that of the QMA and adapted to the Lebanese context.^6^

Half of the respondents had a high level of confidence in doctors, and the medical profession was at the top of health professions. Half of the sample found that doctors were competent, and 39% found them effective. For the Lebanese interviewed, the essential qualities of a doctor are competency and efficiency (50% each). These results were better than those obtained in Canada.^7^ UK findings suggest that social interaction was a predictor of overall satisfaction with primary care.^8^ Modifications in primary health care that allow for more positive interactions between patients and their health care providers can serve to strengthen patient trust in doctors.

Additionally, 48% of the surveyed doctors perceived themselves as leading health professionals compared to pharmacists and nurses regarding the level of the population's full confidence. The majority of doctors (68%) found that they contribute more to patient health than any other health professional, while only 35% of the general population surveyed had the same opinion. The authors assumed that doctors' surrounding conditions under which they perform their daily work influence their self-perceptions.

According to 68% of respondents, being a doctor is a vocation and reflects a commitment to society; the doctors' answers showed similar results. Freidson's conflict theory states that both doctors and patients come from various social and cultural backgrounds.^9^ This social status influences their perceptions and awareness, and the same phenomenon can have a different meaning, be interpreted differently, and take on a different significance. More in-depth studies are needed to understand the societal perspective towards a doctor's job.

Furthermore, 58% of doctors believe that the treatment decision should be discussed with the patient, whereas 40% thought that it was up to the doctor alone to decide on the treatment; this aligns with the answers of the general population surveyed. The four primary models of the doctor-patient relationship explored by Emanuel and colleagues comprise the paternalistic (doctors determine the treatment), the informative (patient choose the treatment), the interpretive (doctor assist patient in deciding), and the deliberative (conversation between the doctor and the patient to identify the best treatment) models.^10^ There was no perfect model to follow; however, a recent study concluded that the deliberative model requires two-way communication between the patient and the doctor. A compassionate manner in addressing encourages mutual decision-making. It enables the doctor to respond to the patient's wishes and values, and the patient to comprehend the knowledge that the doctor provides.^11^

Our analysis has a range of limitations. As a pilot study, the overall sample size was small. As a cross-sectional analysis, information bias, recall failure, and over or under evaluation of essential assessed criteria could exist. Additionally, there might be a disparity in this survey evaluation since there is a difference between patients who visit the doctor once and those who visit him regularly.

The gap between the Lebanese general population and doctors seems to be large, unlike in the Quebec study; however, Lebanese people recognize doctors' contribution to patients' health but have moderate confidence in them. Given these limitations, this study showed valuable findings and may allow further analysis. Further studies should explore the reasons behind the patients' mild trust in their doctors and assess ways to reshape a stronger relationship. It was clear that the image of the medical profession is tarnished, with population education needed in this regard to be able to assume its responsibilities. It is necessary to solicit the medical faculties to include pre and post-doctoral courses to enhance communication between patients and doctors and ensure a professional approach to subsequently reduce the gap of perceptions between doctors and patients. The future of the Lebanese health care system depends on the profession's commitment to reclaim the lost ground, restore the medical profession to its halo, and renew its commitment to society.

### Acknowledgements

The authors would also like to thank Science PRO sarl, Lebanon for their contribution to medical writing.

### Conflict of Interest

The authors declare that they have no conflict of interest.
